# *Hippobosca equina* L. (Hippoboscidae: Hippobosca)—An Old Enemy as an Emerging Threat in the Palearctic Zone

**DOI:** 10.3390/ijerph192416978

**Published:** 2022-12-17

**Authors:** Weronika Maślanko, Ewelina Szwaj, Michał Gazda, Katarzyna Bartosik

**Affiliations:** 1 Department of Animal Ethology and Wildlife Management, University of Life Sciences in Lublin, Akademicka 13 St., 20-950 Lublin, Poland; 2 Ignacy Jan Paderewski Primary School Number 43 in Lublin, Śliwińskiego 5 St., 20-861 Lublin, Poland; 3 Puławy Forest District, Żyrzyńska 8 St., 24-100 Puławy, Poland; 4 Department of Biology and Parasitology, Faculty of Health Sciences, Medical University of Lublin, Radziwiłłowska 11 St., 20-080 Lublin, Poland

**Keywords:** *Hippobosca equina*, Hippoboscidae, hippoboscid, ectoparasite, forest fly, ked, louse fly, parasitic arthropods

## Abstract

Arthropods of the Hippoboscoidea superfamily are parasites of animals from various systematic groups. Mass appearances of these insects and their attacks on people are increasingly being recorded. Their parasitism has a negative effect on host well-being, as it causes feelings of agitation and irritation as well as skin itching and damage. It may result in weight loss and development of diseases in the long-term perspective. Parasites can be a potential epidemiological threat for their hosts as well. One of such parasites is a cosmopolitan species of the Hippoboscinae subfamily—*Hippobosca equina*. Studies have confirmed the presence of *Corynebacterium pseudotuberculosis equi*, *Bartonella* spp., and *Anaplasma* spp. in the organism of these insects. The frequency of anaphylactic reactions caused by *H. equina* attacks has been increasing. The aim of the present paper was to summarize the up-to-date knowledge of *Hippobosca equina* Linnaeus, 1758 due to its significance in medical and veterinary sciences as a potential vector of pathogens. Given the increasing expansion of ectoparasites, mainly related to climate change, ensuring animal welfare and human health is a priority.

## 1. Introduction

Arthropods of the Hippoboscoidea superfamily are ectoparasites of animals from various systematic groups. During zootechnical care or veterinary service, farmers or breeders can be attacked by these nuisance dipteran insects. People that constantly or frequently stay in the natural environment for recreational or professional reasons (e.g., researchers, employees of national parks or forest districts, border guard units, members of hunting associations, etc.) are exposed to the risk of dangerous zoonotic diseases. Our observations from natural and semi-natural habitats and recent reports include the occurrence of these species in cities, which suggests their expansion towards ecosystems undergoing strong anthropogenic transformations [1,2]. Awareness of the consequences of ectoparasite attacks should be one of the main targets of public health systems, especially in the context of the lockdowns imposed during the COVID-19 pandemic, after which people intensified outdoor activities in nature.

The aim of the present paper was to summarize available knowledge of *Hippobosca equina* Linnaeus, 1758 due to its significance in medical and veterinary sciences, and its potential role in pathogen transmission to hosts. Given the increasing expansion of ectoparasites, mainly associated with climate change, ensuring animal welfare and protection of human health is a priority.

The motivation to compile the present review was the authors’ experience of constant exposure to attacks by this ectoparasite in spring and summer 2021 and 2022 in eastern Poland. Moreover, it seems that *Hippobosca equina* is in general not a common object of research, which is evidenced by the inconsiderable number of papers focused on this species. 

## 2. Characteristics of the Species

**Figure 1:**
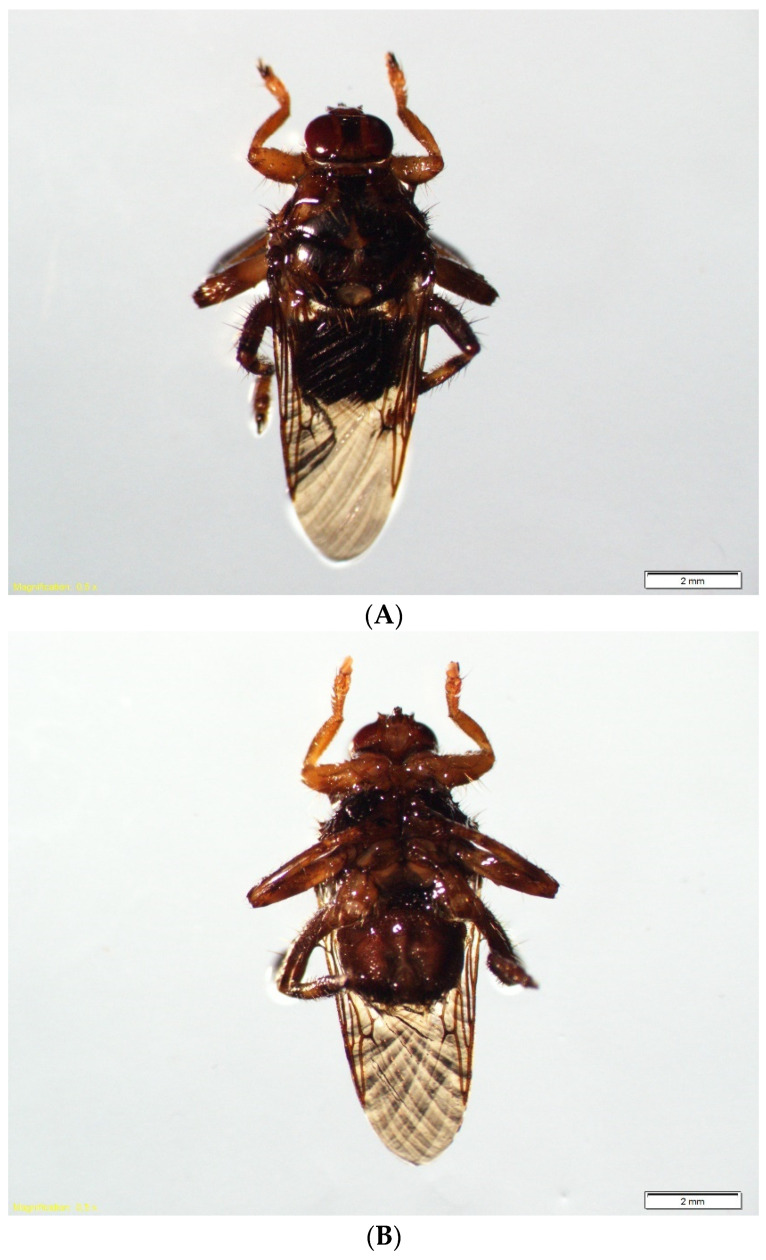
*Hippobosca equina* male: (**A**) dorsal view; (**B**) ventral view (photos by Weronika Maślanko).

*Hippobosca equina* Linnaeus, 1758 (Hippoboscidae: *Hippobosca*) is the earliest described species of the genus (Figure 1). There are seven known species in the genus, i.e., *H. equina* L., 1758, *Hippobosca fulva* Austen 1912, *Hippobosca longipennis* Fabricius, 1805, *Hippobosca camelina* Leach, 1817, *Hippobosca hirsuta* Austen, 1911, *Hippobosca rufipes* von Olfers, 1816, and *Hippobosca variegata* Megerle, 1817.

The studied hippoboscid is commonly known as the forest fly, as well as flat or iron fly, due to the strong dorso-ventral flattening of its chitin exoskeleton [3]. It is an obligate hematophagous ectoparasite of many vertebrate species. 

*Hippobosca equina* is a cosmopolitan species [4] with a wide geographical range covering the Palearctic and West Oriental zones [5]. Taxonomic keys for identification of the species have been proposed by many authors in Poland, e.g., Borowiec [6], or in the United Kingdom, e.g., Bequaert [7]. Despite the availability of the keys for detailed identification of the anatomical structures of *H. equina*, a comprehensive analysis of micro-details requires specialized equipment, such as a scanning electron microscope. Some recent articles presenting high-resolution photographs verify and provide details of these anatomical structures [8,9,10,11,12]. 

Although the Central European population size of the analyzed species has been reported to decline, our observations contradict this finding. Some authors claim that the species was once common but is currently disappearing from some areas of Central Europe [13,14]. However, there are no literature reports confirming this thesis on such a large scale as the whole of Central Europe. The common or rare status of the species cannot be inferred from a few and selective investigations. Only available input and output data (such as fauna inventory) allow assessment of the presence or absence of a species. The ‘rare’ status assigned to a species by some authors may be associated with the lack of experts that could potentially settle the question.

Finding the host is the key to survival for each ectoparasite feeding on a host’s bodily fluids or covering elements. *H. equina* is a temporary parasite of one host; after blood ingestion, it leaves the feeding site and moves to another host or onto the ground where it deposits third instar larvae (pre-pupae) [15,16,17]. The species is evolutionarily specialized through morphological and physiological adaptations to the parasitic lifestyle, e.g., claws on the legs, piercing-sucking mouthparts for ingestion of blood from several to over ten times per day [3], or the prognathous head, which allows it to adhere to the host firmly [11]. The body length of *H. equina* is 7.0–9.0 millimeters, whereas wings length is 6.5–8 millimeters [6]. The insect’s legs are long but strong, widely spaced, with a structure of the foot that allows it to hold on to a single hair [18,19] and climb and attach well to the host [20]. The last segment of the five-segmented foot is long and ends in very strong bifurcate or trifurcate claws. Such a structure allows the insect to move in each direction with extraordinary dexterity and speed [21].

Moreover, the latest research [12] has indicated that the host seeking process in *H. equina* involves various stimuli perceived mainly by specific sensory structures on the antennae, which are the essential olfactory organs. The researchers made a particularly interesting discovery that will be briefly discussed below. The antenna of *Hippobosca equina* exhibits an extraordinary morphological adaptation: it is located in the antennal socket with only the dorsal surface exposed externally, and is covered entirely by microtrichia with the exception of a small area on the top. The distal part of the segment bears three mechanosensory bristles. Additionally, a small furcate arista protrudes in the ventral region of the hollow wallpapered by a dense covering of microtrichia. The flagellum surface is covered by a reticulated cuticle bearing short microtrichia. These microtrichia are shorter and have a wider base than those in *Pseudolynchia canariensis*. However, similar to the cuticular pattern in the pigeon fly, there are several sensilla inside the sensory pits on the dorsolateral region. Coeloconic grooved sensilla, which are almost always sunken in the sensory pits, are mainly spread in the proximal part of the flagellum, while multiporous basiconic sensilla are located around the arista.

Based on the analysis of the specific structure of antennas in *H. equina*, Andeani and co-authors [12] have proposed an interesting hypothesis. They have suggested that the localization of the hosts at medium and long distances by winged adult hippoboscids is mainly facilitated by sensilla; nevertheless, this complex process involves visual stimuli as well. Since there are only few basiconic multiporous sensilla with reduced abundance of wall pores along the shaft, they probably play a role in the host localization at medium-short distances.

The reproduction of *H. equina* is characterized by adenotrophic viviparity and complete metamorphosis (holometabolism). Mating takes place on the host, and stage I, II, and III larvae hatch and develop in the female’s oviduct. The larvae have no head and mouth hooks, and their esophagus acts as a pump. They feed on the secretion produced by secretory uterine glands in the oviduct. The female deposits the first stage-III weakly motile larva, which is ready to pupate. The pupation process lasts ca. 1 h. The pupa is covered by a hardened exuvium that was not discarded at molting. Before the end of the growing season in the temperate climate zone, pupae can begin overwintering, e.g., in wall cracks or on the ground, until the ambient temperature rises. The duration of imago development is variable, which is mainly determined by ambient temperature. At favorable temperatures, the process takes up to 30 days. Mass hatching of adult insects has been observed to take place on hot and humid days. Females deposit 10–15 larvae during their life cycle. In temperate climates, usually one generation develops per year. After emerging from the pupal case, the imago starts host-seeking activity [3,6,19]. 

The presence of adult hippoboscid flies in the environment exhibits strong seasonality in some areas [19]. In temperate climate regions, the species have a seasonal summer pattern during the warmer season from May to October [22,23], as confirmed by research showing that the maximum infestation extensiveness by *H. equina* occurred on July 28 in mares (80%) and on July 26 (90%) and August 16 (70%) in foals [15]. The extensiveness of invasion of the species decreased towards the end of the period, i.e., it decreased from 53.3 to 93.3% in June-July to about 27% in September in young stallions and from over 92% in July and August to 30.8% in September in young mares. In turn, in Saudi Arabia or Israel, the species occurs throughout the year [24,25]. Climate change, resulting in a greater number of days with warmer temperatures, may indicate that mass occurrences of this species will be recorded more frequently. *Hippobosca equina* individuals are active on sunny, warm, or even hot days with high humidity. As shown in some research [3,15], *H. equina* is the most active in the air temperature range of 25–30°C; however, they prefer windless days ([26], own observations). They fly poorly but move on the skin quite fast, causing an unpleasant feeling of tickling and irritation (own observations). The fly may call to mind a spider with wings. *H. equina* specimens are difficult to catch on human or horse skin, as they move fast, but are easier to collect from dog fur [21]. 

*Hippobosca equina* individuals are not good flyers in terms of covering long distances; hence, their parasitism on farm animals does not facilitate natural expansion (migration is only possible through, e.g., sale, transfer, or introduction of infested livestock). In turn, at least partial migration of the ectoparasite is provided by its wild-living animal hosts [27]. 

The described species is easy to identify even without a microscope due to the characteristic wing venation (nearly all veins are darkly pigmented, conspicuous, with a transverse non-pigmented vein) and yellow-milky spots and stripes on the head and thorax. This was described in detail by Bequaert [7] as a pale median patch, extending as a line across the mesoscutum and onto the prescutum, pale humeral borders of the prescutum and the apico-lateral corners of the mesoscutum, mid, and hind tibiae with a pale median band. 

The studied species moves with a decisive, purposeful, error-free movement directly towards the selected target, in contrast to the mosquito, which circulates around the subject before sitting down. In the literature, *H. equina* specimens were described as agile and fast flyers ([3,15], own observations). In contrast to the majority species of Lipopteninae, *H. equina* stays winged throughout the lifespan; nevertheless, it is a rather short-distance flyer, staying close to its site of emergence and waiting for a potential host. 

So far, we have not observed any massive attack of *H. equina*. Our observations indicate that the insects attack rather singly. The largest number of individuals observing the same moving object was three specimens. In comparison, a massive attack by approximately 30 individuals of *Lipoptena cervi* L., 1758 has been observed (own observations). 

## 3. Species Distribution in the World 

In general, the distribution of *H. equina* is associated with the Palearctic and West Oriental zones, although there are some exceptions, as the species has been recorded in Nigeria [28], in the Afrotropical zone. In turn, in Saudi Arabia [25], which is crossed halfway by the border between the Palearctic and Afrotropical zones, *H. equina* was reported to originate in the southern part of the country, belonging to the Afrotropical zone.

The distribution of the studied species confirmed by both scientific papers and/or checklists is shown in Table 1.

The occurrence of *H. equina* recorded in Nigeria (although it was the lowest number of ectoparasites caught in only one published study) seemed extremely interesting and inspired investigations of the presence of the species in the other parts of the Afrotropical zone beyond its official geographical range. However, research conducted so far in Senegal [62] and the Republic of South Africa [63] did not prove the presence of any species from the *Hippobosca* genus, whereas some other species from the genus were identified in Kenya [64] and Madagascar [65].

In Europe, there are no official data from scientific papers and/or no national checklists of invertebrates in some countries, e.g., Iceland, Switzerland, Austria, Serbia, Albania, or Ukraine. This may be associated with the fact that the species has not been observed and identified in these countries or has not been investigated; however, this does not imply that the species does not occur in these regions. Therefore, to present a complete picture of the occurrence of *H. equina*, biodiversity databases available on the internet were used to check any observations in the countries mentioned above and in other regions. Countries where the species was observed and confirmed, in a majority of cases by photographs, are compiled in Table 2.

According to the author of the Polish taxonomic key [6], *H. equina* was introduced unintentionally with horses to Africa, the Oceania islands (Fiji, New Caledonia, New Hebrides), and the Maluku Islands. Although the species distribution in some African countries has been confirmed by scientific papers, the occurrence of the species on islands specified by Borowiec has not been confirmed in the available literature. In turn, the GBIF database contains four reports on the presence of the species on New Caledonia islands, but the date of the observation is not provided. Three of these specimens were deposited in the Australian Museum collection.

## 4. Hosts 

**Figure 2:**
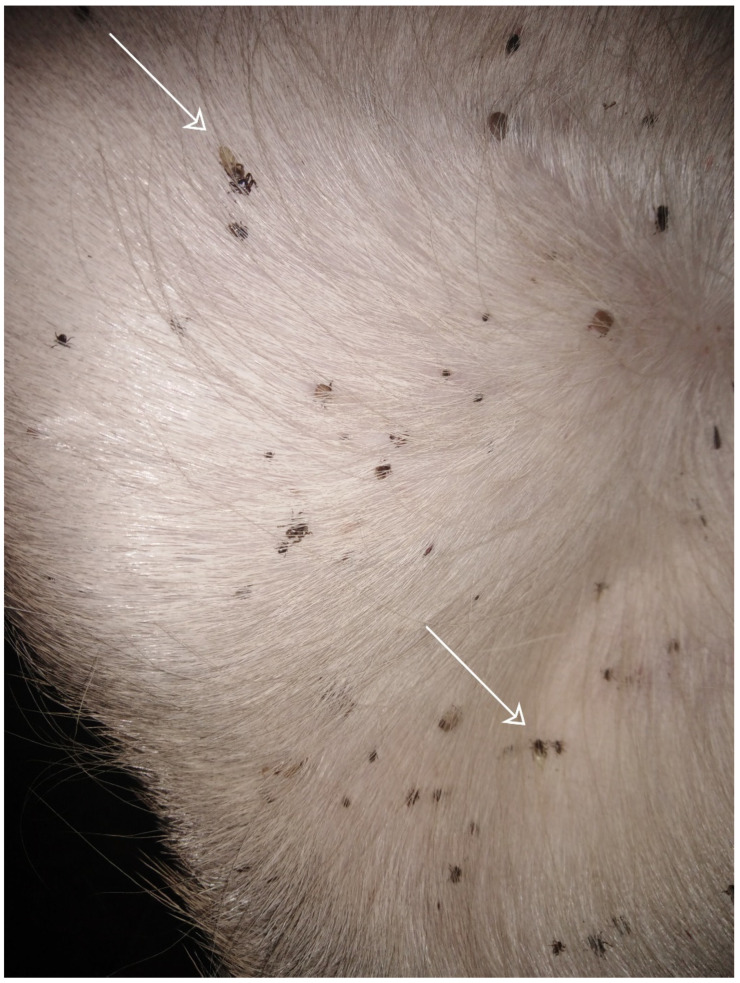
*Hippobosca equina* specimens (arrows) found in groin on red deer calf (photo by Weronika Maślanko).

The hosts of *Hippobosca equina* are warm-blooded animals [66]. Horses and cattle are considered the primary hosts [33]. However, as suggested by Kowal et al. [19], the primary host of the species in Europe is deer, mainly the red deer *Cervus elaphus* L., 1758 (Figure 2). The discrepant views on the role of some animal species as primary hosts are probably caused by the differences in the host occurrence related to geographical regions. On the one hand, the literature and taxonomic keys report that the species is mainly observed on Equidae (horses, donkeys) [57,67], e.g., feral horses [68] or primitive Polish horses *Equus caballus gmelini* [3,15], which corresponds with the word “*equina*” suggesting that horses are in general the primary host. On the other hand, the common name “forest fly” suggests host species living in forest habitats, which suggests, e.g., deer.

Other *H. equina* hosts include camels, rabbits [69], hares, moose, roe deer, grey herons [34,70], northern goshawks [71], goats [49,52], pigeons [20,72], and even dogs [3,24]. Some of the literature from the 1970s reported that the species was also found on *Bison bonasus* L. 1758 in Poland [4]; however, this was not confirmed by any similar reports, e.g., [73,74]. An interesting fact, rarely mentioned in the literature, is that *H. equina* survived and bred on guinea pigs in laboratory conditions in Egypt [75]. This may prove the low host specificity of this species, which is certainly another successful adaptation to the parasitic lifestyle. Soliman et al. [20] highlighted the fact that, due to its low host specificity, *H. equina* poses a threat of disease agents transmission between different host species. 

In terms of the host–parasite association, *H. equina* has lower specificity than different hippoboscids, e.g., *Lipoptena cervi* or *Melophagus ovinus* L., 1758, as evidenced by the presence of wings throughout its lifespan [4], which facilitate migration between host individuals or even between host species. This indicates that *H. equina* is less specific in host selection, which was also reported by Fois et al. [49]. Especially in the present era of anthropopressure and environmental transformation, the possibility of parasitizing many different farmed, companion, or wild animal hosts living in different habitats increases the chances of survival or even expansion of this species. Fois and co-authors [49] have found that *H. equina* specimens can be transferred by humans into any type of habitat (urban, rural or natural).

Although humans are accidental hosts of *H. equina*, our observations and the current medical literature provides new reports on incidents of human infestations by this species [76,77]. However, the literature data concerning this subject are still scarce. Disregard or misidentification of such bites as attacks by other arthropods falsifies the full picture of the risks and consequences of *H. equina* parasitism. Unfortunately, the knowledge of this species and public awareness of its potential of severe infestations is rather insufficient. This may change, however, as even dogs living in cities are attacked by these insects [78]; hence, the interest in this issue will probably increase. 

Kowal et al. [19] suggested that dogs staying or living in forest areas are especially exposed to attacks by Hippoboscidae. A dog attacked by *H. equina* is immensely focused and determined to get rid of the insect from the fur (own observations). Noteworthily, dogs living in cities can be attacked by *H. equina* as well. Recent studies have shown that *H. equina* accounted for 17.2% of all keds identified on dogs living in cities. In general, keds choose female dogs and dogs aged below one most frequently [78]. The species has a tendency to attack young animals, which was confirmed by Romaniuk et al. [15,16,26] in a study on horses. Particularly interesting was the identification of *Hippobosca longipennis* for the first time in Poland. This non-native species in Central Europe typically infests dogs. In the research conducted by Sokół and Gałęcki [78] the species even constituted 45% of all collected keds. 

A common site of attachment of *H. equina* in horses is the sensitive skin around the anus and perineum and the inner surface of the thighs, under the lower part of the vulvar labia in fillies, and in uncoated areas above the anus and often between the thighs above the testicles in stallions [15]. The insect can be found attached to the skin between the tail and ischial tuberosities in cattle. In turn, the species is commonly observed on and under the wings, on the body, and on the tail in pigeons [3,20]. Romaniuk and co-authors [15] reported that, in occasional cases, single insects appeared on horse buttocks or body sides. After movement of the tail, they changed their feeding site and moved onto a different part of the body of the same host or flew away to another host. The same behavior of *H. equina* was observed on dogs [own observations], in which the neck, groin, back, and head were the most common sites of infestation by *Hippobosca* spp. [78]. 

It seems that the reproductive potential of *H. equina* specimens parasitizing occasional hosts has not been fully explored to date. Apart from the study on guinea pigs mentioned previously, no information on other species is available. It has been suggested that, due to the low host specificity [20], the insect can reproduce after feeding on different hosts, depending on the area of occurrence. However, in the case of another subfamily of Hippoboscidae, i.e., Lipopteninae, it has been found that humans are attacked as hosts for species of the genus *Lipoptena* spp. Mistakenly, as the insects cannot reproduce on this host [79]. For this reason, humans are even considered an ecological trap [80,81] as the parasite loses its wings after settling on the host. Furthermore, deer keds are characterized by different reproduction performance on hosts, e.g., reproduction on *Capreolus capreolus* L., 1758 has proved to be successful only in the western range of Fennoscandia, whereas in the eastern range, successful offspring production has been observed only once [82]. There are no similar data about *H. equina*. The final confirmation of the above-mentioned information requires a detailed analysis of the reproductive behavior of *H. equina*. 

Data on the prevalence and intensity of *H. equina* infestations in the world are rather limited. The only studies were conducted in Poland, e.g., [3,16], and Egypt [20]. Researchers [3] studying the prevalence of *H. equina* in horses in Poland reported the highest prevalence from mid-June to the end of July at an average air temperature of 21 °C. In turn, the highest prevalence of *H. equina* in Egypt was recorded from mid-June to the end of August [20]. The prevalence of *H. equina* infestation in feral horses was 80.6%. The mean number of *H. equina* specimens per infested animal was 3.2 (range 1–10) [68], whereas the abundance reported in other studies was estimated at 5–100 in cattle, 1–80 in buffalo, 1–25 in horses, 5–60 in donkeys, and 2–6 in pigeons [20]. 

Research on primitive Polish horses demonstrated that the extensiveness and intensity of infestation fluctuated between 23 and 38.5% and 0.6 and 2.2 in mares, 10 and 30% and 1.0 in sucking colts. These values were 40–80% and 2.1–9 in 1.5–2-year-old mares and 53.3–86.7% and 3.8–6.5 in stallions, respectively. This indicates that the intensity of invasion infestation in 2005 was not significant, as it did not exceed nine insects on one horse in July [15]. In 2012 and 2013, other researchers carrying out investigations in the same region as Romaniuk et al. 2007 [15] reported the highest intensity of infestation in working geldings (28 to 34 insects per horse), 1.5-year-old colts (10 to 16 insects), and mares with foals (4 to 14 insects) [3].

According to data obtained from research carried out on horses, *H. equina* was found on all examined hosts [15,68]. In some horses, *H. equina* was the only ectoparasite species found [57], which may suggest that the insect was the most common ectoparasite. These results may confirm that horses are an important host for *H. equina.*

The predicted increase in the abundance of *H. equina*, as well as the range expansion, probably depends on an increase in host density. Such a relationship was observed in the case of *L. cervi* in Finland [83,84]. In the context of these phenomena in *H. equina*, climate change may affect the growth of wild host populations. In Europe, Artiodactyla and, above all, *Cervus elaphus* L. are the primary hosts of this species [19]. Indeed, in Europe, Asia and Africa, the occurrence ranges of both *C. elaphus* and *H. equina* largely overlap. We frequently observed the mass occurrence of *H. equina*, in eastern Poland, where a large red deer population has been recorded in recent years. As shown by the data provided by the Central Statistical Office, in 2013–2019, the red deer population in Lubelskie Province alone increased by 30.26%. The constant rise in the number of red deer and their higher density increases the probability of reproductive success for *H. equina* [85,86].

Warm weather increases the probability of host acquisition by winged *Lipoptena cervi* long into late autumn. Changes in temperature and precipitation also affect many life traits of hematophagous ectoparasites, including host seeking behavior [87]. In the above-mentioned representatives of Hippoboscidae, the survival of juveniles during the off-host stage is also facilitated by higher temperatures. Moreover, numerous adaptations to unfavorable environmental conditions can be observed in Hippoboscidae during the non-parasitic phase of the life cycle [88,89,90]. Predictions for an increase in ambient temperature will probably contribute to the territorial expansion of ectothermic species belonging to Hippoboscidae [84]. 

Due to the lack of this type of research on *H. equina*, we cite studies conducted by specialists from Scandinavia, on other representatives of the Hippoboscidae, and arthropod vectors with similar biology and ecology. Therefore, we presume that, factors affecting the distribution and abundance of Hippoboscidae representatives may be alike to a large extent.

Variable conditions in habitats influence not only the vectors and hosts (often reservoir animals), but also transmission of vector-borne pathogens [91].

## 5. Medical and Veterinary Importance of *Hippobosca equina*

*Hippobosca equina* parasitism has a negative effect on host well-being, as it causes feelings of agitation and irritation as well as skin itching and damage. The evident consequences of the species parasitism in animal hosts include anxiety manifested in intensive tail lashing and frequent scraping against posts or tree trunks, as well as skin inflammation, loss of body weight, and decreasing of the quality of host skin, hair, and fleece [4,15]. In general, forest flies influence animals’ behavior, and they may become more active and nervous or suffer from colic [78]. In the long run, it may lead to the development of diseases [15]. 

**Figure 3:**
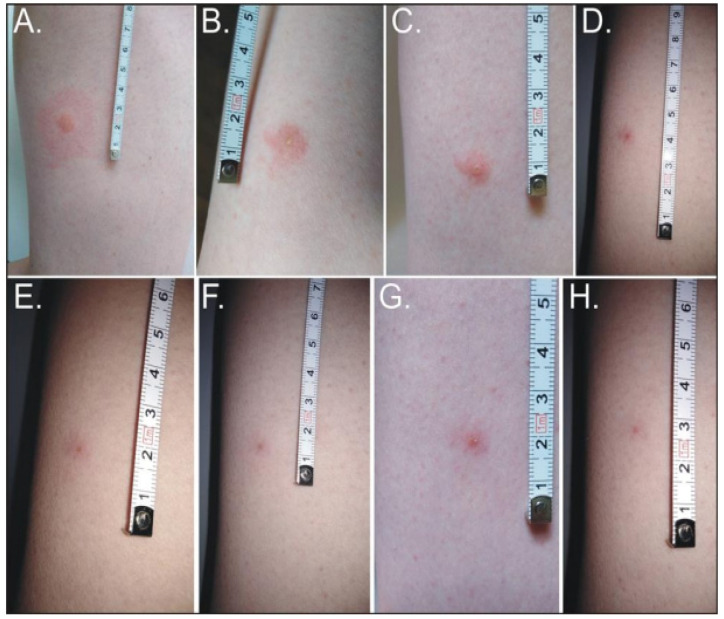
Evolution of skin lesions after *Hippobosca equina* bite. (**A**,**B**) Multiple painful urticarial pink papules on an erythematous base on the thigh: (**A**) regular 19 h after the bite; (**B**) 40 h after the bite; (**C**) excoriated erythematous papule with self-limited dermatitis 64 h after the bite; (**D**) 99 h after the bite; (**E**) 131 h after the bite; (**F**) 196 h after the bite; (**G**) 260 h after the bite, (**H**) residual hyperpigmented papule 19 days after the bite (photos by Weronika Maślanko).

In humans, the bite of the forest fly is barely noticeable, or painless, but an increasing burning sensation with erythema may start to develop after a few hours (Figure 3). To date, the available medical literature has reported only four cases of human infestations by this insect species [76,77,92,93]. Anaphylactic reactions, e.g., erythema, nausea, facial angioedema, vomiting, dyspnea, dizziness, or tachycardia, developed in the infested patients.

As demonstrated by the latest research, *H. equina* is considered a vector of pathogens. This role is particularly important, as the insect attacks different hosts (within the same species and between different species). Unlike other hippoboscid flies, *H. equina* is a permanently full-winged species. Soliman et al. [20] highlighted the link between the low host specificity of *H. equina* and the danger of disease agents transmission between different host species. As shown by this researcher, *H. equina* plays such a role in both biological and mechanical transmission between different animal species. Similar conclusions were formulated by Arafa et al. [94], who indicated *H. equina* as a mechanical and biological vector of Oedematous Skin Disease (OSD) in buffaloes. This endemic disease in Egypt is characterized by diffused skin swellings in the limbs, abdominal region, and dewlap. Another symptom of the disease is the inflammation and enlargement of regional drainage lymph nodes [95,96]. *Hippobosca equina* is involved in the horizontal transmission of* Corynebacterium pseudotuberculosis equi* bacteria. These pathogens were detected in 76.9% of *H. equina* adults feeding on animals examined and 68.5% of skin lesion samples taken from these animals [94]. In addition, Arafa et al. reported transstadial transmission of *C. pseudotuberculosis equi* in *H. equina* (vertical transmission) [94]. The occurrence of vertical transmission is one of the arguments for the vector competence of *H. equina*. As a result of this phenomenon, the vector can also serve as a pathogen reservoir. Hence, the forest fly was recognized as the main transmitter of the causative agent to buffaloes [97]. On a global scale, this bacterium is pathogenic to ruminants and horses but rarely to humans. It causes losses in livestock and, consequently, economic losses for breeders. Nevertheless, research results inspired scientists to devise a vaccine. Syame et al. [98] tested two preparations: recombinant-bacterin and toxoid-bacterin vaccine applied to hairless body areas, such as axillary or groin folds. Although both vaccines showed equal vaccinal efficacy, they differed in the stimulation of the immune system to produce anti-PLD IgG (higher titers in the case of the recombinant-bacterin vaccine). The authors did not investigate the exact duration of immunity, but it was recommended that the vaccination against OSD should be performed at the end of February to cover summer months, i.e., the period of the highest *H. equina* activity.

Research conducted in Saxony showed that 82% of the *H. equina* specimens studied were positive for *Bartonella* spp. [40]. Similar results were reported by Boucheikhchoukh et al. [9], who found that 75.86% of *H. equina* collected from horses were *Bartonella*-positive. It was found in the same research that 13.79% of the collected *H. equina* specimens were infected by pathogenic bacteria from the genus *Wolbachia*. In turn, Halos and co-authors [31] detected *Bartonella chomelii* and/or *Bartonella schoenbuchensis* in all twelve *H. equina* specimens collected from cattle in different parts of France, but none of the six *H. equina* collected from horses was positive. Abdullah et al. [57] confirmed the presence of both *Borrelia* sp. and *Anaplasma* sp. in *H. equina* specimens collected from horses in Egypt. 

To date, there is no evidence that *H. equina* is a vector of other pathogens, e.g., *Rickettsia* spp. Abdullah and co-authors [57] attempted to detect species belonging to piroplasmida, *Rickettsia* sp., *Coxiella burnetii*, and *Bartonella* sp. pathogens in this insect species. All 105 studied *H. equina* specimens were found to be free from these pathogens. Interestingly, Selmi et al. [56] reported that horses infested by *H. equina* in Tunisia exhibited a higher *Anaplasma* spp. infection rate than other, non-infested animals. All these bacteria cause animal and human diseases. Notably, the mere detection of a given pathogen in *H. equina* does not mean the arthropod is its competent vector; only that the assumption that it can serve as its reservoir is probable.

The potential role of the forest fly as a vector of various pathogens is also important, given the possibility of unintentional introduction of the species with the host. Since *Hippobosca equina* feeds on the blood of a wide range of hosts, and adults of these insects take multiple blood meals during their lifespan, protection against its attacks seems to be relevant. However, the most commonly used repellents, e.g., DEET (N,N-diethyl-3-methylbenzamide), have recently raised concerns related not only to their safety but also to the increasing insect resistance [99,100]. Therefore, essential oils (EOs) have been considered an alternative to synthetic repellents due to their safety and toxicity profiles to humans, animals, and the environment [101,102,103]. High repellence of plant compounds against various parasitic arthropods has been confirmed [99,100,101]; nonetheless, there is insufficient research on repellents as protective measures against *Hippobosca equina* attacks. Khater et al. demonstrated the repellent activity of camphor (*Cinnamomum camphora*), onion (*Allium cepa*), chamomile (*Matricaria chamomilla*), and peppermint (*Mentha piperita*) EOs toward dipteran ectoparasites infesting water buffaloes in Egypt [104]. The doses of EOs applied by pour-on treatment were 1.4, 2.9, 3.4, and 3.6 mL kg^−1^ b.w., respectively (discriminating dose 2.5 l). According to the study, all tested EOs repelled *Hippobosca equina* significantly for six days post-treatment. These results are promising because the researchers did not observe any side effects in humans or treated animals. The adverse effects usually related to the application of EOs are mild. However, severe toxic reactions have been noted, including neurotoxicity, bronchial hyperactivity, and hepatotoxicity [105]. Therefore, in the case of using EOs-based protective measures, precautions should be taken, especially in humans and animals with respiratory diseases, skin disorders, and allergies.

## 6. Conclusions

The analysis of the current distribution of the species worldwide carried out in this review indicates that it was also found outside the originally assumed range of two biogeographic zones (Palearctic and West Oriental zones), i.e., in the Afrotropical and Australasian zones. Although these were only single records (three and four in each zone, respectively), they may indicate a potential or actual spread of this species and, consequently, a threat to animals and humans in other parts of the world. The observations from the Philippines and New Caledonia date back to the 20th century (the latter was presented by Borowiec [6]), whereas there are no data on the current abundance of the species, which suggests the need to explore this issue comprehensively.

As shown by Boucheikhchoukh and co-authors [9], forest flies feeding on humans may often not be discerned; hence, subsequent skin manifestations or disease symptoms are not associated with the attack by these insects. Additionally, due to the insufficient knowledge of disease agents transmission, the insects are not perceived as an epidemiological threat. Therefore, studies of the biology and ecology of this species, as well as the medical consequences of forest fly bites, should be continued.

## Figures and Tables

**Table 1:** Countries with recorded occurrence of *Hippobosca equina* L., 1758.

	Observations of *Hippobosca equina* L. 1758	
Zone	Country	Data Source
**Palearctic**	Azores, Portugal	[29]
	Spain	[30]
	France	[31,32]
	Great Britain	[33,34,35]
	Belgium	[36]
	The Netherlands	[37]
	Germany	[38,39,40]
	Poland	[6,19,41], own observations 2021, 2022
	Norway	[42]
	Sweden	[43]
	Finland	[44]
	Estonia	[45]
	Latvia	[46]
	Lithuania	[47]
	Hungary	[39]
	Croatia	[39,48]
	North Macedonia	[39]
	Bosnia and Herzegovina	[39]
	Continental Italy	[10]
	Sardinia, Italy	[49]
	Slovakia	[14,39]
	Belarus	[50]
	Romania	[51]
	Bulgaria	[52]
	The Cyclades, Greece	[53]
	Azerbaijan	[54]
	Armenia	[54]
	Iran	[54,55]
	Algeria	[9]
	Tunisia	[56]
	Egypt	[57]
	Israel	[24]
	Jeju Island, Republic of Korea	[58,59]
**West Oriental**	India	[60,61]
**Afrotropical**	Nigeria	[28]

**Table 2:** Records confirming *Hippobosca equina* occurrence according to the internet biodiversity databases (only in countries where the occurrence of *H. equina* was not confirmed by scientific papers or national checklists).

	Observations of *Hippobosca equina* L. 1758	
Name of Database	Country	Month and Year
**GBIF** ^1,^*	**Palearctic zone**	
	Continental Portugal	01.2015. x 164; 05.2018. x 2; 06.2018. x 3; 10.2018. x 2; 07.2020; 04.2021.; 04.2022.; 05.2022., 06.2022.
	Slovenia	08.2015. x 2
	Austria	06.2017.; 05.2018.; 03.2020., 05.2022.
	Sweden	10.2003.; 06.2012.; 06.2014.; 07.2015. x 4; 05.2016.; 07.2016.; 08.2017.; 06.2020.; 07.2020. x 3, 05.2022.; 06.2022.
	Continental Greece	07.2012.; 08.2018.; 07.2020.
	The Republic of Turkey	03.2022.
	Kazakhstan	09.2020.
	**Oriental zone**	
	The Philippines	05.1911. ** x 4
	**Australasian zone**	
	New Caledonia	no data ** x 4
	**Afrotropical zone**	
	Mozambique	04.1928.; 01.1930.; 07.1968.
**iNaturalist** ^2,^***	Tenerife, Spain	09.2016.
	Portugal	06.2020. x 2; 04.2022.; 05.2022.; 06.2022.
	Austria	05.2018; 03.2020.; 05.2022. x 2
	The Czech Republic	07.2016.
	Corsica, France	09.2012.; 07.2020.
	Continental Greece	07.2020.
	Lesbos	08.2018.
	North Aegean, Greece	08.2022.; 09.2022.
	Romania	09.2019.
	The Republic of Turkey	03.2022.
	Ukraine	07.2020.
	Kazakhstan	09.2020.
**Fauna Europaea** ^3^	The Canary Islands	no data
	Ireland	
	Norway	
	Denmark	
	Switzerland	
	Sicily, Italy	
	The Czech Republic	
	Crete, Greece	
	The Dodecanese Islands, Greece	
**PESI** ^4^	The Balearic Islands, SpainSwitzerland The Czech republic	no data
	Denmark	

^1^ Global Biodiversity Information Facility (https://www.gbif.org/ (accessed on 13 October 2022)); 1155 observations; ^2^ iNaturalist (https://www.inaturalist.org/ (accessed on 13 October 2022)); 201 observations; ^3^ https://fauna-eu.org/ (accessed on 13 October 2022); 29 observations; ^4^ Pan-European Species-directories Infrastructure (http://www.eu-nomen.eu/ (accessed on 13 October 2022)); * records from the Palearctic zone deposited in this database only since 2000 were selected; ** specimens preserved in collections; *** the only database providing photographs from each observation.

## Data Availability

Data supporting reported results can be found here: Global Biodiversity Information Facility (https://www.gbif.org/ (accessed on 13 October 2022)); iNaturalist (https://www.inaturalist.org/ (accessed on 13 October 2022)); (https://fauna-eu.org/ (accessed on 13 October 2022)); Pan-European Species-directories Infrastructure (http://www.eu-nomen.eu/ (accessed on 13 October 2022)).

## References

[1] Kowal J., Kornaś S., Nosal P., Wajdzik M., Basiaga M., Lesiak M. (2015). Parasite infections in red deer *Cervus elaphus* from Krakow area, southern Poland. Ann. Parasitol..

[2] Gałęcki R., Xuan X., Bakuła T. (2022). Migration of deer keds to urban agglomerations—A case study. Ann Parasitol..

[3] Sokół R., Michalski M. (2015). Occurrence of *Hippobosca equina* in Polish primitive horses during the grazing season. Ann. Parasitol..

[4] Kadulski S. (1970). Materiały do znajomości Hippoboscidae (Diptera-Pupipara) ssaków użytkowych Polski. Wiadomości Parazytol..

[5] Oboňa J., Krišovský P., Hromada M. (2019). Short-term faunistic sampling of Louse flies (Diptera: Hippoboscidae) from Drienovec Bird Ringing Station, Slovakia. Biodivers. Environ..

[6] Borowiec L. (1984). Klucze do Oznaczania Owadów Polski. Część XXVIII. Muchówki—Diptera. Zeszyt 77, Wpleszczowate—Hippoboscidae.

[7] Bequaert J. (1930). Notes on Hippoboscidae. 2 The Subfamily Hippoboscinae. Psyche.

[8] Zhang D., Liu X.H., Li X.Y., Cao J., Chu H.J., Li K. (2015). Ultrastructural investigation of antennae in three cutaneous myiasis flies: *Melophagus ovinus*, *Hippobosca equina*, and *Hippobosca longipennis* (Diptera: Hippoboscidae). Parasitol. Res..

[9] Boucheikhchoukh M., Mechouk N., Benakhla A., Raoult D., Parola P. (2019). Molecular evidence of bacteria in *Melophagus ovinus* sheep keds and *Hippobosca equina* forest flies collected from sheep and horses innortheastern Algeria. Comp. Immunol. Microbiol. Infect. Dis..

[10] Andreani A., Sacchetti P., Belcari A. (2020). Evolutionary adaptations in four hippoboscid fly species belonging to three different subfamilies. Med. Vet. Entomol..

[11] Andreani A., Sacchetti P., Belcari A. (2020). Keds and Bat Flies (Hippoboscidae, Nycteribiidae and Streblidae). Reference Module in Biomedical Sciences, Encyklopedia of Infection and Immunity.

[12] Andreani A., Belcari A., Sacchetti P., Romani R. (2022). Antennal Morphology and Fine Structure of Flagellar Sensilla in Hippoboscid Flies with Special Reference to *Lipoptena fortisetosa* (Diptera: Hippoboscidae). Insects.

[13] Jedlička L., Stloukalová V., Baláž D., Marhold K., Urban P. (2001). Červený (ekosozoologický) zoznam dvojkrídlovcov (Diptera) Slovenska. Červený Zoznam Rastlín Aživočíchov Slovenska.

[14] Oboňa J., Oldřich S., Greš S., Heřman P., Manko P., Roháček J., Šestáková A., Šlapák J.J., Hromada M. (2019). A revised annotated checklist of louse flies (Diptera, Hippoboscidae) from Slovakia. ZooKeys.

[15] Romaniuk K., Gad K., Kiszka W. (2007). Występowanie muchówki *Hippobosca equina* u koników polskich. Med. Weter..

[16] Johnsen P. (1948). Notes on the Danish louse-flies (Diptera: Hippoboscidae). Entomol. Medd..

[17] Mehlhorn H. (2016). Hippoboscidae. Encyclopedia of Parasitology.

[18] Haarløv N. (1964). Life Cycle and Distribution Pattern of *Lipoptena cervi* (L.) (Dipt., Hippobosc.) on Danish Deer. Oikos.

[19] Kowal J., Nosal P., Kornaś S., Wajdzik M., Matysek M., Basiaga M. (2016). Różnorodność i znaczenie muchówek z rodziny narzępikowatych—Pasożytów jeleniowatych. Med. Weter..

[20] Soliman S.M., Attia M.M., Al-Harbi M.S., Saad A.M., El-Saadony M.T., Salem H.M. (2022). Low host specificity of *Hippobosca equina* infestation in different domestic animals and pigeon. Saudi J. Biol. Sci..

[21] Kulczycki W. (1892). Owady Pasorzytujące u Ludzi i Zwierząt Domowych.

[22] Pfadt R.E., Roberts I.H., Bram R.A. (1978). Louse flies (family Hippoboscidae). Surveillance and Collection of Arthropods of Veterinary Importance.

[23] Zittra C., Schoener E.R., Wagner R., Heddergott M., Duscher G.G., Fuehrer H.-P. (2020). Unnoticed arrival of two dipteran species in Austria: The synanthropic moth fly *Clogmia albipunctata* (Williston, 1893) and the parasitic bird louse fly *Ornithoica turdi* (Olivier in Latreille, 1811). Parasitol. Res..

[24] Wallach A., Shanas U., Mumcuoglu K.Y., Inbar M. (2008). Ectoparasites on Reintroduced Roe Deer *Capreolus capreolus* in Israel. J. Wildl. Dis..

[25] El-Hawagry M.S., Khalil M.W., Sharaf M.R., Fadl H.H., Aldawood A.S. (2013). A preliminary study on the insect fauna of Al-Baha Province, Saudi Arabia, with descriptions of two new species. ZooKeys.

[26] Romaniuk K. (2004). Występowanie muchówek u krów i koników polskich przebywających na pastwisku. Med. Weter..

[27] Łabędzki A. (2012). Krwiopijcy z lasów i łąk. Wiedza Życie.

[28] Abubakar B.A., Falmata K., ThankGod O.E., Abdulmalik A., Ali M. (2018). Survey of flies (order: Diptera) of Medical and Veterinary importance infesting livestock in Maiduguri, Borno state, Nigeria. J. Sci. Agric..

[29] Kameneva E.P., Borges P.A.V., Costa A., Cunha R., Gabriel R., Gonçalves V., Martins A.F., Melo I., Parente M., Raposeiro P., Rodrigues P. (2010). B—Diptera—Ulidiidae. 11. Lista dos Artrópodes (Arthropoda).

[30] Vázquez L., Dacal V., Pato F.J., Díaz P., Painceira A., Fernández G., Morrondo P., Diez-Baños P. (2010). Ectoparásitos presentes en corzos (*Capreolus capreolus*) de Galicia (NO España). Galemys.

[31] Halos L., Jamal T., Maillard R., Girard B., Guillot J., Chomel B., Vayssier-Taussat M., Boulouis H.J. (2004). Role of Hippoboscidae flies as potential vectors of *Bartonella* spp. infecting wild and domestic ruminants. Appl. Environ. Microbiol. J..

[32] Liénard E., Salem A., Grisez C., Prévot F., Bergeaud J.P., Franc M., Gottstein B., Alzieu J.P., Lagalisse Y., Jacquiet P. (2011). A longitudinal study of *Besnoitia besnoiti* infections and seasonal abundance of Stomoxys calcitrans in a dairy cattle farm of southwest France. Vet. Parasitol..

[33] Hutson A.M., Fitton M.G. (1984). Keds, flat-flies and bat-flies. Diptera, Hippoboscidae and Nycteribiidae. Handbooks for the Identification of British Insects.

[34] Turner C.R., Mann D.J. (2004). Recent observations of *Hippobosca equina* L. (Diptera: Hippoboscidae) in South Devon. Br. J. Entomol. Nat. Hist..

[35] Chandler P.J. (2021). An Update of the 1998 Checklist of Diptera of the British Isles.

[36] Grootaert P., De Bruyn L., De Meyer M. (1991). Catalogue of the Diptera of Belgium.

[37] Beuk P.L.T. (2001). Family Hippoboscidae. In Checklist of the Diptera of the Netherlands. https://diptera-info.nl/news.php?fam=Hippoboscidae.

[38] Jentzsch M., Müller J., Frank D., Schnitter P. (2016). Lausfliegen (Diptera: Hippoboscidae). Pflanzen und Tiere in Sachsen-Anhalt.

[39] Jentzsch M., Knauthe C.H. (2019). Louse fly collections of institutes and museums in Saxony (Diptera, Hippoboscidae). Entomol. Nachr. Ber..

[40] Schröter S., Freick M., Vogt I., Jentzsch M. (2020). Lausfliegen (Hippoboscidae: Diptera) als Vektoren für Bakterien mit Zoonosepotential bei Säugetieren. Beiträge Jagd Wildforschung.

[41] Draber-Mońko A., Durska E., Klasa A., Kownacki A., Krzemiński W., Razowski J. (1991). 28. Diptera—Muchówki. Checklist of Animals of Poland.

[42] Siebke H. (1877). Catalogum Dipterorum Continentem. Programmatis Nomine Edidit Universitas Regia Fredericiana. http://www.entomologi.no/journals/enumeratio/Enumeratio_04_1877.pdf.

[43] Andersson H. (1985). De svenska lusflugorna. The Swedish louse-flies (Diptera: Hippoboscidae). Entomol. Tidskr..

[44] Pohjoismäki J., Kahanpää J. (2014). Checklist of the superfamilies Oestroidea and Hippoboscoidea of Finland (Insecta, Diptera). ZooKeys.

[45] Teder T., Tammaru T., Chinery M. (2005). Insects of Britain and Western Europe. Euroopa Putukad.

[46] Karpa A. (2008). Catalogue of Latvian Flies (Diptera: Brachycera). Latv. Entomol..

[47] Pakalniškis S., Bernotienė R., Lutovinovas E., Petrašiūnas A., Podėnas S., Rimšaitė J., Sæther O.A., Spungis V. (2006). Checklist Of Lithuanian Diptera. New Rare Lith. Insect Species.

[48] Trilar T., Krčmar S. (2005). Contribution to the knowledge of louse flies of Croatia (Diptera: Hippoboscidae). Nat. Croat..

[49] Fois F., Mereu Piras P., Cappai S., Cillo D., Culurgioni J., Deiana A.M., Mandas L., Rolesu S. (2012). Contribution to the knowledge of Diptera Hippoboscidae in Sardinia. Mappe Parassitol..

[50] Гембiцкi А.С., Давыдава Г.П., Гембiцкi А.С. (1983). Весцi Акадэмii навук БССР. Крывасысучыя Двухкрылыя Басейна Вoзера Нарач.

[51] Billeter S.A., Levy M.G., Chomel B.B., Breitschwerdt E.B. (2008). Vector transmission of Bartonella species with emphasis on the potential for tick transmission. Med. Vet. Entomol..

[52] Prelezov P., Nizamov N. (2020). A case of multiple mixed invasion with ectoparasites in goats. Tradit. Mod. Vet. Med..

[53] Alexiou S., Gavalas I. (2017). Calyptrate flies (Diptera) of Cyclades, Greece—I. Families Calliphoridae, Hippoboscidae, Oestridae, Polleniidae, Rhiniidae, Rhinophoridae and Scathophagidae. Studia Dipterol..

[54] Nartshuk E.P., Oboňa J. (2019). The distribution of genus Hippobosca in Transcaucasia. Acta Musei Sil. Sci. Nat..

[55] Sazmand A., Bahari A., Papi S., Otranto D. (2020). Parasitic diseases of equids in Iran (1931–2020): A literature review. Parasites Vectors.

[56] Selmi R., Dhibi M., Ben Said M., Ben Yahia H., Abdelaali H., Ameur H., Baccouche S., Gritli A., Mhadhbi M. (2019). Evidence of natural infections with *Trypanosoma*, *Anaplasma* and *Babesia* spp. in military livestock fromTunisia. Trop. Biomed..

[57] Abdullah H.H.A.M., Aboelsoued D., Farag T.K., Abdel-Shafy S., Abdel Megeed K.N., Parola P., Raoult D., Mediannikov O. (2022). Molecular characterization of some equine vector-borne diseases and associated arthropods in Egypt. Acta Trop..

[58] Seok D.M. (1970). The Insect Fauna of the Island Quelpart.

[59] Kim H.C., Chong S.T., Chae J.-S., Lee H., Klein T.A., Suh S.J., Rueda L.M. (2010). New Record of *Lipoptena cervi* and Updated Checklist of the Louse Flies (Diptera: Hippoboscidae) of the Republic of Korea. J. Med. Entomol..

[60] Rani P.A.M., Coleman G.T., Irwin P.J., Traub R.J. (2011). *Hippobosca longipennis*—A potential intermediate host of a species of Acanthocheilonema in dogs in northern India. Parasit Vectors.

[61] Maity A., Naskar A., Mitra S., Chakraborty A., Hazra S., Banerjee D. (2014). Checklist of Indian Louse Flies (Insecta: Diptera: Hippoboscidae). Zsi Publ..

[62] Sychra O., Literák I., Najer T., Čapek M., Koubek P., Procházka P. (2010). Chewing lice (Insecta: Phthiraptera) from estrildid finches (Aves: Passeriformes: Estrildidae) and louse-flies (Insecta: Diptera: Hippoboscidae) from birds in Senegal, with descriptions of three new species of the genus Brueelia. Zootaxa.

[63] Sychra O., Halajian A., Engelbrecht D., Symes C.T., Oschadleus H.D., de Swardt D.H., Papousek I. (2020). Louse-flies (Diptera: Hippoboscidae) of birds from South Africa: Prevalence and diversity. Afr. Entomol..

[64] Oboňa T.J., Zeegers T., Wamiti W., Njoroge N. (2016). Additions to the Checklist of the Louse Flies (Diptera: Hippoboscidae) of Kenya. Afr. Entomol..

[65] Rachola N., Goodman S.M., Robert V. (2011). The Hippoboscidae (Insecta: Diptera) from Madagascar, with new records from the “Parc National de Midongy Befotaka”. Parasite.

[66] Kazimierczak K., Górski P. (2007). Narzępikowate w Polsce—biologia i znaczenie. Życie Weter..

[67] Bezerra-Santos M.A., Otranto D. (2020). Keds, the enigmatic flies and their role as vectors of pathogens. Acta Trop..

[68] Dik B., Ceylan O., Ceylan C., Tekindal M.A., Semassel A., Sönmez G., Ekici Ö.D. (2020). Ectoparasites of feral horses (*Equus ferus caballus* (Linnaeus., 1758)) on Karadağ Mountain, Karaman, Turkey. J. Parasit. Dis..

[69] Maa T.C. (1969). A revised checklist and concise host index of Hippoboscidae (Diptera). Pac. Insects Monogr..

[70] Olafsson E. (1985). A heron carrying louse flies to Iceland. Bliki.

[71] Krištofík J., Štefan P. (1980). K poznaniu čelade Hippoboscidae (Diptera) na Slovensku. Biológia Bratisl..

[72] Salem H.M., Yehia N., Al-Otaibi S., El-Shehawi A.M., Elrys A.A.M.E., El-Saadony M.T., Attia M.M. (2021). The prevalence and intensity of external parasites in domestic pigeons (Columba livia domestica) in Egypt with special reference to the role of deltamethrin as insecticidal agent. Saudi J. Biol. Sci..

[73] Karbowiak G., Demiaszkiewicz A.W., Pyziel A.M., Wita I., Moskwa B., Werszko J., Bień J., Goździk K., Lachowicz J., Cabaj W. (2014). The parasitic fauna of the European bison (*Bison bonasus*) (Linnaeus, 1758) and their impact on the conservation. Part 1. The summarising list of parasites noted. Acta Parasitol..

[74] Karbowiak G., Demiaszkiewicz A.W., Pyziel A.M., Wita I., Moskwa B., Werszko J., Bień J., Goździk K., Lachowicz J., Cabaj W. (2014). The parasitic fauna of the European bison (*Bison bonasus*) (Linnaeus, 1758) and their impact on the conservation. Part 2. The structure and changes over time. Acta Parasitol..

[75] Hafez M., Hilali M., Fouda M. (1977). Biological studies on *Hippobosca equina* L. (Diptera: Hippoboscidae) infesting domestic animals in Egypt. Entomology.

[76] Quercia O., Emiliani F., Foschi F.G., Stefanini G.F. (2005). Anaphylactic reaction after *Hippobosca equina* bite. Eur. Ann. Allergy Cinical Immunol..

[77] Matito A., Bartolomé-Zavala B., Álvarez-Twose I., Sánchez-Matas I., Escribano L. (2010). IgE-mediated anaphylaxis to *Hippobosca equina* in a patient with systemic mastocytosis. Allergy.

[78] Sokół R., Gałęcki R. (2017). Prevalence of keds on city dogs in central Poland. Med. Vet. Entomol..

[79] Hodžić A., Omeragić J., Alić A., Jažić A., Zuko A. (2012). *Lipoptena cervi* (Diptera: Hippoboscidae) in Roe deer (*Capreolus capreolus*). Veterinaria.

[80] Kaunisto S., Korter R., Härkönen L., Härkönen S., Ylönen H., Laaksonen S. (2009). New bedding site examination-based method to analyse deer ked (*Lipoptena cervi*) infection in cervids. Parasitol. Res..

[81] Robertson B.A., Hutto R.L. (2006). A framework for understanding ecological traps and an evaluation of existing evidence. Ecology.

[82] Härkönen L., Kaitala A., Canning-Clode J. (2015). Host Dynamics and Ectoparasite Life Histories of Invasive And Non-Invasive Deer Ked Populations. Biological Invasions in Changing Ecosystems.

[83] Meier C.M., Bonte D., Kaitala A., Ovaskainen O. (2014). Invasion rate of deer ked depends on spatiotemporal variation in host density. Bull. Entomol. Res..

[84] Kynkäänniemi S.M., Kortet R., Laaksonen S. (2020). Range expansion and reproduction of the ectoparasitic deer ked (*Lipoptena cervi*) in its novel host, the Arctic reindeer (*Rangifer tarandus tarandus*), in Finland. Parasitol. Res..

[85] Central Statistical Office (2014). Environment, Statistical Information and Elaborations, 2014.

[86] Central Statistical Office (2019). Environment, Statistical Information and Elaborations, 2019.

[87] Mysterud A., Madslien K., Herland A., Viljugrein H., Ytrehus B. (2016). Phenology of deer ked (*Lipoptena cervi*) host-seeking flight activity and its relationship with prevailing autumn weather. Parasites Vectors.

[88] Kaunisto S., Ylonen H., Kortet R. (2015). Passive sinking into the snow as possible survival strategy during the off-host stage in an insect ectoparasite. Folia Parasitol..

[89] Nieminen P., Käkelä R., Paakkonen T., Halonen T., Mustonen A.M. (2013). Fatty acid modifications during autumnal cold-hardening in an obligatory ectoparasite, the deer ked *(Lipoptena cervi*). J. Insect Physiol..

[90] Härkönen L., Hurme E., Kaitala A. (2013). Unexpected seasonal variation in offspring size and performance in a viviparous ectoparasite. Parasitology.

[91] El-Sayed A., Kamel M. (2020). Climatic changes and their role in emergence and re-emergence of diseases. Environ. Sci. Pollut. Res. Int..

[92] Vidal C., Armisén M., Bartolomé B., Rodriguez V., Luna I. (2007). Anaphylaxis to *Hippobosca equina* (louse fly). Eur. Ann. Allergy Cinical Immunol..

[93] Decastello A., Farkas R. (2010). Anaphylactic reaction following forest fly (*Hippobosca equina*) bite: A human case. Clin. Exp. Med. J..

[94] Arafa M.I., Hamouda S.M., Rateb H.Z., Abdel-Hafeez M.M., Aamer A.A. (2019). Oedematous Skin Disease (OSD) transmission among buffaloes. Global Journal of Medical Research: G. Vet. Sci. Vet. Med..

[95] Selim S.A. (2001). Review Oedematous Skin Disease of Buffalo in Egypt. J. Vet. Med. Ser. B.

[96] Moussa I.M., Ali M.S., Hessain A.M., Kabli S.A., Hemeg H.A., Selim S.A. (2016). Vaccination against Corynebacterium pseudotuberculosis infections controlling caseous lymphadenitis (CLA) and oedematous skin disease. Saudi J. Biol. Sci..

[97] Ghoneim M.A., Mousa W.M., Ibrahim A.K., Amin A.S., Khafagy A., Selim S.A. (2001). Role of *Hippobosca equina* as a transmitter of C. pseudotuberculosis among buffaloes by PCR and Dot blot hydridization. Egypt. Vet. Med. Assoc..

[98] Syame S.M., EL-Hewairy H.M., Selim S.A. (2008). Protection of Buffaloes Against Oedematous Skin Disease by Recombinent-bacterin and Toxoid-bacterin Vaccines. Glob. Vet..

[99] Benelli G., Pavela R. (2018). Repellence of essential oils and selected compounds against ticks—A systematic review. Acta Trop..

[100] Benelli G., Pavela R. (2018). Beyond mosquitoes—Essential oil toxicity and repellency against bloodsucking insects. Ind. Crops Prod..

[101] Lee M.Y. (2018). Essential Oils as Repellents against Arthropods. BioMed Res. Int..

[102] Khanikor B., Parida P., Yadav R.N.S., Bora D. (2013). Comparative mode of action of some terpene compounds against octopamine receptor and acetyl cholinesterase of mosquito and human system by the help of homology modeling and Docking studies. J. Appl. Pharm. Sci..

[103] Nerio L.S., Olivero-Verbel J., Stashenko E. (2010). Repellent activity of essential oils: A review. Bioresour. Technol..

[104] Khater H.F., Ramadan M.Y., El-Madawy R.S. (2009). Lousicidal, ovicidal and repellent efficacy of some essential oils against lice and flies infesting water buffaloes in Egypt. Vet. Parasitol..

[105] Ramsey J.T., Shropshire B.C., Nagy T.R., Chambers K.D., Li Y., Korach K.S. (2020). Essential Oils and Health. Yale J. Biol. Med..

